# Subacute cutaneous lupus erythematosus triggered by sintilimab: a case report

**DOI:** 10.3389/fimmu.2025.1544312

**Published:** 2025-04-24

**Authors:** Chenxi Zhang, Xiaoying Zhang, Jiali Liang, Yifei Liu, Jialong Chen, Qi Wang, Changxing Li

**Affiliations:** ^1^ Department of Dermatology, Southern Hospital, Southern Medical University, Guangzhou, China; ^2^ Department of Dermatology, the Tenth Affiliated Hospital, Southern Medical University (Dongguan People’s Hospital), Dongguan, China

**Keywords:** sintilimab, PD-1 inhibitor, immune-related adverse events, subacute cutaneous lupus erythematosus, immunotherapy

## Abstract

Immune checkpoint inhibitors (ICIs) have become a standard treatment for various cancers, but their use is often associated with immune-related adverse events (irAEs), including cutaneous irAEs (cirAEs). Here, we report a rare case of subacute cutaneous lupus erythematosus (SCLE) induced by sintilimab, a PD-1 inhibitor, in a 30-year-old woman undergoing neoadjuvant chemo-immunotherapy for gastric cancer. The patient presented with erythema, macules, papules, and vesicles, with positive ANA (108U/mL) and strongly positive anti-SSA/Ro. After discontinuation of sintilimab and treatment with corticosteroids, hydroxychloroquine, and intravenous immunoglobulin (IVIG), her symptoms improved. This case represents the first reported instance of drug-induced lupus caused by sintilimab and emphasizes the importance of distinguishing between paraneoplastic lupus and ICI-induced lupus.

## Introduction

Immune checkpoint inhibitors (ICIs), including T-lymphocyte-associated antigen-4 (CTLA-4), anti-programmed cell death protein-1 (PD-1) antibodies, and anti-programmed death-ligand 1 (PD-L1) inhibitors, have been widely used in the treatment of tumors over the past decade (1). They effectively curb the immune evasion mechanisms of tumor cells by activating T cells to enhance their ability to attack tumor cells. However, stimulating an antitumor immune response also leads to the occurrence of immune-related adverse events (irAEs) (1). Among these, cutaneous irAEs (cirAEs) are the most common, such as maculopapular eruption, pruritus, and vitiligo-like lesions (2). ICI-induced subacute cutaneous lupus erythematosus (SCLE) is rare. Although there have been several reports of PD-1 inhibitor-induced SCLE, such as pembrolizumab and nivolumab, no cases have been associated with sintilimab (3). Here, we report a case of sintilimab-induced SCLE in a 30-year-old woman with gastrointestinal malignancies, providing insights into the understanding and management of this rare cirAE.

## Case report

**Figure 1:**
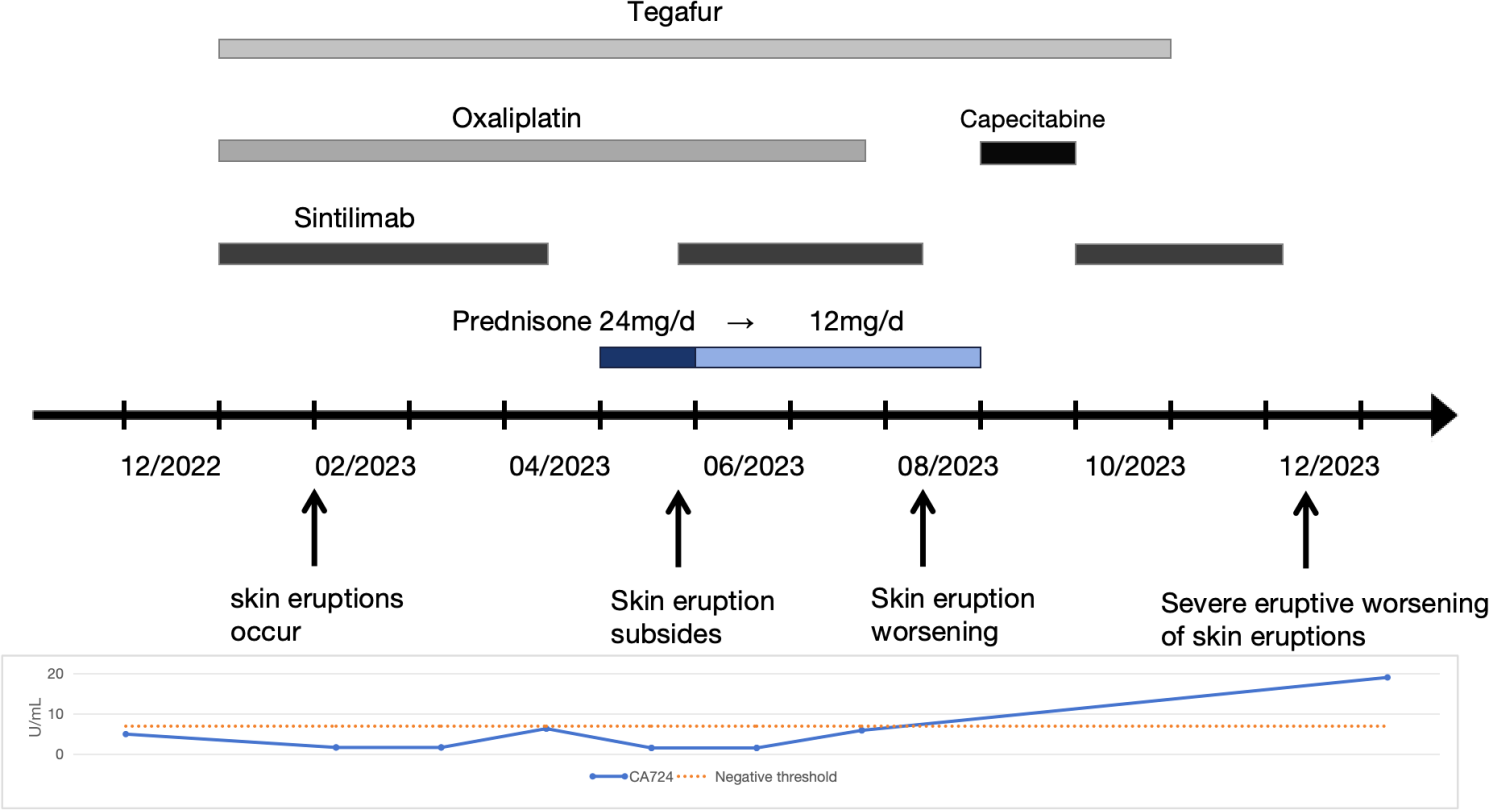
Therapeutic and clinical timeline. A chronological overview of the patient’s treatment regimen, symptom progression, and relevant laboratory findings. Oxaliplatin (100 mg every 3 weeks), Tegafur (40 mg twice daily for 14 days, followed by a 7-day interval), Sintilimab (200 mg every 3 weeks), and Capecitabine (1 g twice daily).

**Figure 2:**
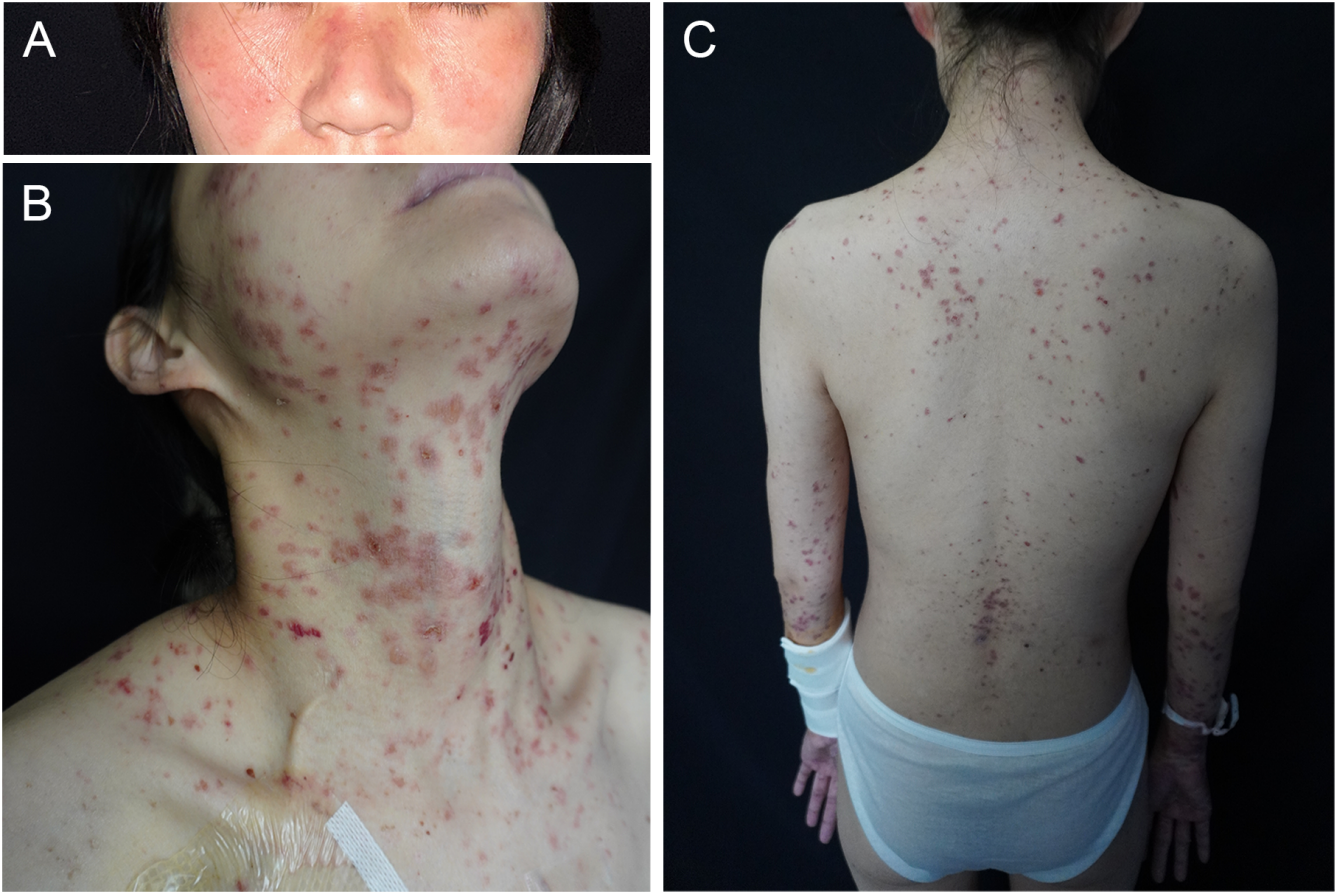
Clinical images. **(A)** Butterfly-shaped facial erythema, **(B)** severely pruritic erythematous macules, papules, and vesicles on sun-exposed areas (V-area of the neck, arms), and **(C)** on the back.

**Figure 3:**
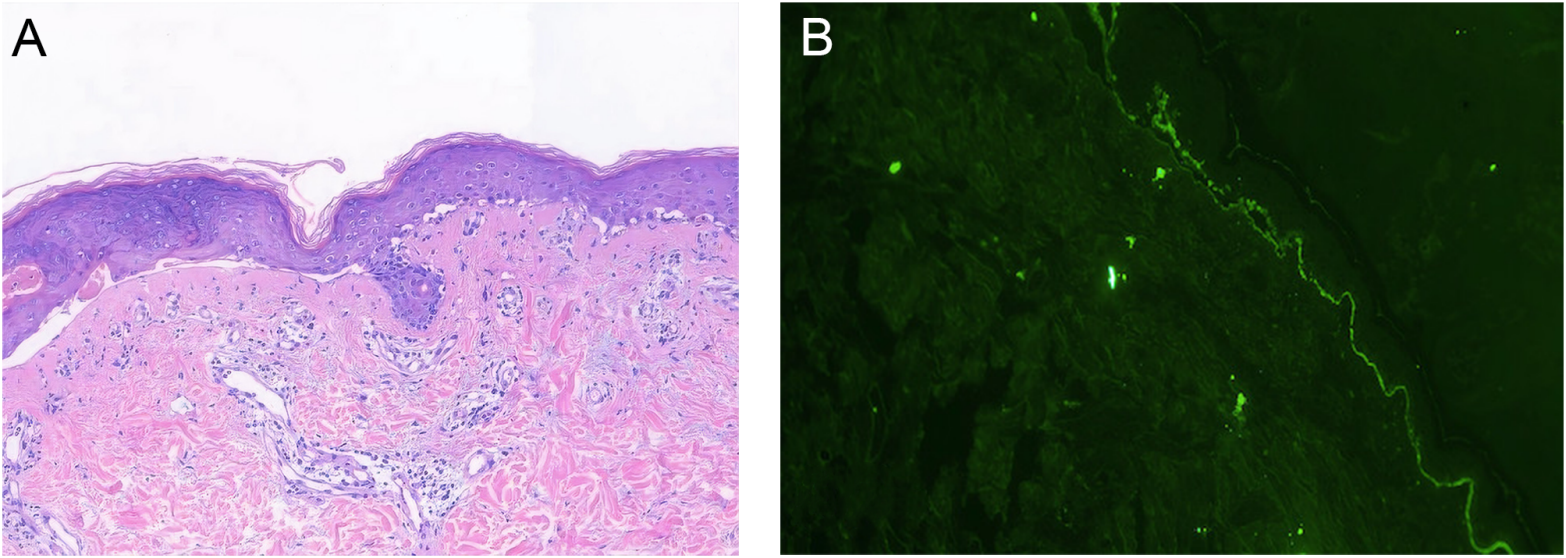
Histopathology. **(A)** Skin biopsy, HES staining, ×10: Vacuolation of the basal keratinocytes, with the presence of blisters between the dermis and epidermis. Infiltration of numerous lymphocytes, plasma cells, and scattered neutrophils was observed around the superficial dermal blood vessels. **(B)** Direct immunofluorescence (DIF) examination, ×10: C3 deposition was noted along the basement membrane of the epidermis.

A 30-year-old woman with gastric antral carcinoma was referred to the dermatology department for an 8-month history of severely pruritic, butterfly-shaped facial erythema, photodistributed erythematous macules, scaly papules, and vesicles. One year prior, she had undergone palliative gastrectomy at our hospital, followed by a combination treatment regimen, including oxaliplatin, tegafur, and sintilimab. During the second cycle of the therapy, the patient developed a butterfly-shaped facial erythema, along with erythematous macules and papules on the limbs, accompanied by itching. Therefore, in the fifth cycle of the treatment, sintilimab was discontinued, and oral prednisone was administered (0.5 mg/kg/d). The patient’s rash subsequently improved, allowing for the continuation of sintilimab in the sixth cycle, during which the rash did not improve. After completing the eighth cycle, the patient discontinued prednisone and began the maintenance phase with a regimen of tegafur and sintilimab. Following the first cycle of the therapy, the patient’s rash worsened. The oncologist used tegafur and capecitabine as monotherapy in the second and third cycles, respectively, and the patient’s rash improved slightly. In the fourth, fifth, and sixth cycles, the patient switched to sintilimab monotherapy (**Figure 1**). Five days after the completion of the sixth cycle, the patient’s skin manifestations suddenly worsened, prompting further treatment in the dermatology department (**Figure 2**). The patient was diagnosed with drug-induced, immune-related SCLE, Common Terminology Criteria for Adverse Events version 5.0 (CTCAE v5.0) grade 3. Laboratory tests: Anti-SSA/Ro antibodies (using ELISA) were strongly positive, with the ANA at 108 U/mL, while dsDNA and anti-Sm antibodies were negative. Pathological examination (skin biopsy of the patient**’**s left forearm): There was vacuolation of the basal keratinocytes, with the presence of blisters between the dermis and epidermis. Infiltration of numerous lymphocytes, plasma cells, and scattered neutrophils was observed around the superficial dermal blood vessels. Direct immunofluorescence (DIF) examination: C3 deposition was noted along the basement membrane of the epidermis, with a small amount of IgG deposition around the blood vessels (**Figure 3**). Treatment: ICI therapy was discontinued, and the patient received intravenous methylprednisolone (1–2 mg/kg/d), intravenous immunoglobulin (IVIG) impulse therapy (17.5 g/d, a total of 5 days), and oral hydroxychloroquine (200 mg twice daily). After 4 weeks, the facial erythema of the patient resolved, leaving behind some hyperpigmentation. The erythema on the trunk faded, the blisters flattened, and the exudation from the ulcerated areas decreased, with scabbing beginning to occur. Fifteen days later, the patient was switched to oral capecitabine under the guidance of the oncologist. Two months later, while anti-SSA/Ro antibodies became weakly positive, the ANA titer was below 1:80 and considered negative, and the skin eruptions improved, the malignancy unfortunately progressed. Four months later, the patient was lost to follow-up.

## Discussion

Sintilimab is a PD-1 inhibitor that has demonstrated efficacy in the treatment of gastrointestinal tumors (4). These drugs activate the immune system but may also cause irAEs affecting multiple organs, including the skin, thyroid, gastrointestinal tract, liver, and lungs, with cutaneous reactions being the most prevalent (1). In patients receiving immune checkpoint inhibitors (ICIs), the incidence of cirAEs ranges from 30% to 60%, which is associated with both the type of ICI used and the type of tumor (5).

At present, the combination of tegafur, oxaliplatin, and PD-1 inhibitors is considered a first-line therapeutic regimen for HER-2 negative gastrointestinal cancers. Previous case reports have indicated that 5-fluorouracil can induce drug-induced lupus (6), and combination therapy is associated with a higher incidence of cutaneous adverse reactions than monotherapy (5). Oncologists may employ single-agent therapy to identify sensitizing drugs, as demonstrated by the patient in our case, who showed improvement in skin eruptions after discontinuing sintilimab, only for the eruption to worsen upon re-administration of the drug.

Immune checkpoint inhibitor-induced lupus (ICI-LE) is a rare subtype of cirAEs. Most of the available literature consists of case reports and small case series. ICI-LE is most commonly associated with anti-PD-1/PD-L1 inhibitors (e.g., pembrolizumab and nivolumab) and is less frequently observed with anti-CTLA-4 inhibitors (e.g., ipilimumab) (2). A recent review summarizing 29 cases of different malignancies treated with various ICIs proposed that the average onset of ICI-LE occurred 6.14 months after ICI initiation, with an average resolution time of 2.1 months, and 2 out of 29 (6.8%) cases exhibited refractory symptoms (2). Subacute cutaneous lupus erythematosus (SCLE) represents the predominant clinical phenotype, comprising 86.2% of cases, whereas systemic lupus erythematosus (SLE) is less frequently reported, accounting for 13.7% of cases (2).

PD-1 and its ligands (PD-L1 and PD-L2) are immune-regulatory molecules involved in the pathogenesis of lupus, potentially contributing to the breakdown of immune tolerance and the onset of autoimmunity (7). Their expression is altered in lupus, though the precise consequences of this dysregulation remain unclear. In SLE patients, dysregulation of the PD-1 pathway is associated with T cell activation, abnormal B cell proliferation, and heightened inflammatory responses (7). Two key pathways of interest, the Toll-like receptor (TLR) and type I interferon (IFN) signaling pathways, regulate PD-1 and its ligands through NF-κB and STAT1 activation (7–9). Additionally, abnormalities in TAM receptors (Tyro3, Axl, and Mer) and the aryl hydrocarbon receptor (AHR) may further disrupt the regulation of the PD-1 axis in SLE (7).

Subacute cutaneous lupus erythematosus (SCLE) predominantly presents in two clinical morphologies, annular polycyclic lesions and psoriasiform papulosquamous lesions. Drug-induced SCLE constitutes more than 30% of cases and is characterized by greater skin involvement and atypical presentations, such as bullous or targetoid lesions, which are less frequently observed in idiopathic SCLE (10). This aligns with the skin manifestations observed in our reported case.

SCLE can result not only from drug adverse reactions but also as a paraneoplastic manifestation in cancer patients. In our case, the patient had elevated CA724 levels, highlighting the importance of distinguishing whether SCLE is a paraneoplastic skin manifestation or an irAE caused by ICIs. Paraneoplastic skin manifestations typically appear before the diagnosis of malignancy, serving as an early warning and correlating with poor tumor prognosis (11). Additionally, these manifestations often parallel tumor progression, and in most cases, improvement in skin lupus symptoms is observed with cancer treatment alone (11). In contrast, cirAEs occur several weeks to months after treatment initiation. In addition, a cohort study demonstrated that cirAEs are associated with improved survival in primary malignancies, with a six-month landmark analysis and time-varying Cox proportional hazards models showing a reduced mortality risk in cirAEs patients, particularly in melanoma patients (12). Examples include vitiligo, psoriasis-like rashes, acneiform eruptions, erythema, and isolated pruritus, particularly in melanoma, where vitiligo is associated with a favorable prognosis. This may result from the expansion of antigenic epitopes following melanoma cell death, with activated T cells targeting both tumor cells and melanocytes in normal skin (12). Furthermore, in drug-induced subacute lupus, the titers of SSA/Ro antibodies typically decrease within 8 months after discontinuation of drug treatment (13). Based on the following observations, we diagnosed this case as drug-induced lupus: the patient’s skin eruptions appeared after drug administration; imaging and clinical findings indicated that the malignancy remained stable during both the onset and exacerbation of the skin eruptions; and two months after discharge, although malignancy progressed, the anti-SSA/Ro antibodies became weakly positive, the ANA titer was considered negative, accompanied by an improvement in the skin eruptions.

The American Society of Clinical Oncology (ASCO) has established a grading system for the severity of cirAEs (CTCAE, Common Terminology Criteria for Adverse Events), which classifies them from G1 (Grade 1) to G4 based on body surface area (BSA) involvement and associated symptoms. For G1 to G3 skin irAEs, treatment typically continues without discontinuation, with management focused on topical or oral corticosteroids (1). In contrast, approximately 51.7% of patients with ICI-LE discontinue ICI therapy (3). In our case report, the patient initially developed a mild skin eruption, affecting less than 10% of the BSA and was classified as G1 according to CTCAE criteria. Given its limited severity, the oncologist opted to continue sintilimab treatment. However, following the final administration of sintilimab, the patient experienced a sudden and severe flare-up of the skin eruption, escalating to G3. In accordance with ASCO guidelines and existing case reports, as well as considering the patient’s personal preference, ICI therapy was discontinued.

In conclusion, we report the first documented case of SCLE induced by sintilimab, with a differential diagnosis between paraneoplastic lupus and ICI-induced lupus. The patient’s skin eruptions improved following appropriate intervention. This case highlights the importance of distinguishing immune-related adverse events from paraneoplastic manifestations to ensure accurate diagnosis and management. For patients with ICI-LE who continue ICI therapy, regular follow-up should include monitoring of skin manifestations, autoimmune antibody levels (e.g., ANA, anti-SSA/Ro), and tumor progression. Standardized grading based on CTCAE and timely intervention are critical for optimizing patient outcomes. The mechanisms underlying ICI-LE remain undetermined, necessitating further research to elucidate its pathogenesis and inform future clinical management strategies. The use of immune checkpoint inhibitors has extended survival in cancer patients, providing clinical benefit, but immune-related adverse events can reduce quality of life. Balancing survival and quality of life remains a key objective for oncologists and other specialists.

## Data Availability

The raw data supporting the conclusions of this article will be made available by the authors, without undue reservation.
